# Hospital Festivals and Meetings

**Published:** 1895-04-06

**Authors:** 


### HOSPITAL FESTIVALS AND MEETINGS.

### ROYAL EYE HOSPITAL, SOUTHWARK.

On Wednesday, March 27th, the annual general meeting of the governors of the Royal Eye Hospital, Southwark, was held at the hospital in one of the wards which are at present closed from want of funds. The chair was taken by Professor McHardy. The report of the council for 1894 showed that a large number of new patients had been treated during the year, of whom 490 were admitted as in-patients. The council had been compelled to sell £2,488 of Consols in order to meet expenses, the income of the hospital still proving inadequate. The 1895 festival dinner was announced to take place on June 18th, at which Mr. R. K. Causton, M.P., will preside. The chairman spoke of the reforms already accomplished and in course of completion in ophthalmic hospitals throughout the kingdom, instancing the contemplated changes at Moorfields, at the Royal Westminster Ophthalmic Hospital, and other reconstructions on modern and improved lines at Exeter, Cork, and Sheffield, and commented upon the evident appreciation of the work of the Royal Eye Hospital by the poor and suffering, as shown by their use of its advantages. During the course of the meeting Mr. Henry Irving was unanimously elected a vice-president in recognition of his kindly interest in the hospital.

### NATIONAL HOSPITAL FOR THE PARALYSED AND EPILEPTIC.

A festival dinner in connection with this hospital was held in the Hotel Metropole on Tuesday evening. The Duke of Cambridge presided, and the company assembled was large and representative. There were present among others, Lord Halsbury, Field Marshal Sir Donald Stewart, Admiral Saumarez, General Sir Redvers Buller, Sir J. Russell Reynolds, President of the Royal College of Physicians, Canon Duckworth, Sir J. Crichton-Browne, Mr. Justice Bruce, the Hon. H. Dudley Ryder, and the Hon. W. F. B. Massey-Mainwaring. The dinner was very successful, and cannot fail to widen interest in the important special work which this hospital is doing. Not only so, but the immediate result in the shape of contributions to the funds was substantial. A happy note was struck at the opening, when the announcement was made of a subscription from Her Majesty the Queen of £50, an announcement which was received with loud cheers.

The Duke of Cambridge made an excellent chairman. The Duke, as Canon Duckworth fitly pointed out, has throughout his long career been the warm friend of British charities, and the appeal which he made on behalf of the National Hospital for the Paralysed and Epileptic evinced not only knowledge of the work that is done, but heartfelt sympathy with the sufferers for whose sake the hospital exists. Times had been bad said the Duke, but he hoped they had not been so bad as to make it necessary that an institution that had been doing so much good as that hospital had done should suffer. The population had largely increased, and with this the demand for hospital accommodation—though he feared that hospitals had not increased in the same proportion. In connection with the special work of that hospital, the diseases which were treated at it were now much better understood than they used to be; they required more careful attention than it was possible to give at the older general hospitals. To maintain that hospital, which with its new wing could accommodate 200 patients, a sum of £15,000 per year was needed. Of that sum they could reckon on only £10,000 from investments and subscriptions as "fairly reliable"; and for the balance they had to appeal to the public. Moreover, money was needed to pay for the building of the convalescent home at East Finchley. There was every reason to believe that money given to that hospital would be well expended. There should be no stint in giving to such a useful, valuable, and well-conducted institution, which well deserved their generosity, support, and confidence. He sincerely trusted, for the sake of those who suffered from the terrible afflictions and diseases that made life almost unbearable, that that meeting would have good results.

Lord Halsbury, who replied, said the like of that hospital did not exist in the kingdom. They had patients from all parts of the world. One who desired to remain anonymous, who had been deeply afflicted but had been relieved, had intimated the intention of giving £2,000 to their building fund. (Cheers.) He stated that other hospitals refused to relieve epileptics from whatever diseases they were suffering—(hear, hear)—and the result was that they became like the lepers of old—like pariahs; and for the sake of these he urgently appealed for their sympathy.

On the call of Canon Duckworth, the "Health of the Chairman" was then honoured, and the Duke of Cambridge in reply made an interesting little speech. He had been many years in public life, he said, but he had never forgotten that he was an Englishman from the bottom of his heart. (Hear, hear.) Following the example of the late duke, his father, and the other members of his family, he had taken an interest in the charitable institutions of this country. There was no country in the world that had such magnificent voluntary institutions as this. No doubt other countries were jealous, but we should not mind that. At any rate, we should never let the State do what should come from the heart. (Cheers.)

General Sir Redvers Buller then proposed "The Board of Management," and the Hon. H. Dudley Ryder, treasurer of the hospital, replied.

Sir J. Russell Reynolds proposed "The Medical and Surgical Staff," and gave some interesting reminiscences in connection with the hospital, of the staff of which he himself was at one time a member. He dwelt specially on the valuable educational work that was being done. A school of neurology had been established second to none in England. Dr. Gowers, in acknowledging, mentioned that during the past eight years no fewer than two thousand medical students and medical practitioners had received instruction at the institution.

Sir James Crichton-Browne, who proposed "The Contributors and Visitors," pointed out that at this time this hospital has a special claim on the benevolent. The disastrous waves of influenza which had passed over the land had left behind them a residuum of misery which had reduced the nerve energy of Europe by 20 per cent. For years to come that hospital would be thronged by victims of influenza. The Hon. W. F. B. Massey-Mainwaring acknowledged the toast.

Mr. B. Burford Rawlings, Secretary and General Director of the Hospital, announced subscriptions to the general purposes and building funds, which amounted to a grand total of £4 440. This included £50 from Her Majesty and £2,000 from the anonymous donor referred to in the course of the evening. The announcement, which it was felt fully justified the gathering, was received with cheers.

